# Identification of fish spermatogenic cells through high-throughput immunofluorescence against testis with an antibody set

**DOI:** 10.3389/fendo.2023.1044318

**Published:** 2023-04-03

**Authors:** Ding Ye, Tao Liu, Yongming Li, Yaping Wang, Wei Hu, Zuoyan Zhu, Yonghua Sun

**Affiliations:** ^1^ State Key Laboratory of Freshwater Ecology and Biotechnology, Hubei Hongshan Laboratory, Institute of Hydrobiology, Innovation Academy for Seed Design, Chinese Academy of Sciences, Wuhan, China; ^2^ College of Fisheries and Life Science, Dalian Ocean University, Dalian, China

**Keywords:** Ddx4, Sycp3, Piwil1, PCNA, spermatogonia, spermatocyte

## Abstract

Image-based identification and quantification of different types of spermatogenic cells is of great importance, not only for reproductive studies but also for genetic breeding. Here, we have developed antibodies against spermatogenesis-related proteins in zebrafish (*Danio rerio*), including Ddx4, Piwil1, Sycp3, and Pcna, and a high-throughput method for immunofluorescence analysis of zebrafish testicular sections. By immunofluorescence analysis of zebrafish testes, our results demonstrate that the expression of Ddx4 decreases progressively during spermatogenesis, Piwil1 is strongly expressed in type A spermatogonia and moderately expressed in type B spermatogonia, and Sycp3 has distinct expression patterns in different subtypes of spermatocytes. Additionally, we observed polar expression of Sycp3 and Pcna in primary spermatocytes at the leptotene stage. By a triple staining of Ddx4, Sycp3, and Pcna, different types/subtypes of spermatogenic cells were easily characterized. We further demonstrated the practicality of our antibodies in other fish species, including Chinese rare minnow (*Gobiocypris rarus*), common carp (*Cyprinus carpio*), blunt snout bream (*Megalobrama amblycephala*), rice field eel (*Monopterus albus*) and grass carp (*Ctenopharyngodon idella*). Finally, we proposed an integrated criterion for identifying different types/subtypes of spermatogenic cells in zebrafish and other fishes using this high-throughput immunofluorescence approach based on these antibodies. Therefore, our study provides a simple, practical, and efficient tool for the study of spermatogenesis in fish species.

## 1 Introduction

In vertebrates, gametogenesis, regulated by genetic, endocrine, and environmental factors, gives rise to mature gametes that transmit genetic material and hence phenotypes to subsequent generations by sexual reproduction. Spermatogenesis in fish is a complex process consisting of cell proliferation and differentiation that gives rise to different types of spermatogenic cells, from mitotic spermatogonial stem cells (SSCs) to meiotic spermatocytes and finally to spermatozoa (1, 2). Fish are the most diverse group of vertebrates, and it requires different time for different fish species from fertilization to the appearance of spermatozoa (2, 3). It is about 50 days for zebrafish (*Danio rerio*), one of the most commonly used model animals (4), about 4 months for Chinese rare minnow (*Gobiocypris rarus*), an experimental fish widely used for toxicological study (5), about 5 months for common carp (*Cyprinus carpio*), a worldwide cultivated freshwater species (6, 7), about one year for blunt snout bream (*Megalobrama amblycephala*), a predominant aquaculture species in China (8, 9), about two years for rice field eel (*Monopterus albus*), a commercially important protogynous hermaphroditic fish (10, 11), and about four to six years for grass carp (*Ctenopharyngodon idellus*), a fish contributing the largest aquaculture production in the world (12–14). On the other hand, in the application of surrogate reproduction by spermatogonial transplantation, only undifferentiated SSCs can efficiently differentiate into mature gametes in the host fish (1, 15, 16). Therefore, characterization of different spermatogenic cells and investigation of the underlying cellular events are important not only for fish reproductive research (17–21) but also for germ cell-based genetic breeding such as surrogate reproduction (5, 22–26).

To characterize different spermatogenic cells, histological analysis has been widely used to visualize cell morphological and nuclear characteristics during spermatogenesis (27, 28). However, the underlying molecular and cellular events of spermatogenesis need to be investigated with immunofluorescence staining with specific antibodies against different spermatogenic factors. There are several commonly used markers for studying spermatogenesis, including Ddx4 (DEAD (Asp-Glu-Ala-Asp) box polypeptide 4), Piwil1 (Piwi-like RNA-mediated gene silencing 1), Sycp3 (Synaptonemal complex protein 3) and Pcna (Proliferating cell nuclear antigen). Both Ddx4 and Piwil1 play critical roles in the biogenesis of PIWI-interacting RNAs (piRNAs) and silencing of transposable elements during spermatogenesis (29–32). Cell mitotic proliferation is generally indicated by the expression of Pcna, an essential component of the DNA replication machinery (33–35), while meiotic differentiation of spermatogenic cells is indicated by the expression of Sycp3, an essential element of the synaptonemal complex (36). Previously, these markers have been used selectively in various studies (1, 18, 37), but an integrated analysis of these proteins by immunofluorescence during fish spermatogenesis is lacking.

The procedures of immunofluorescence include sample preparation, sectioning, first antibody incubation, secondary antibody incubation, washing, image acquisition, and data processing. Usually, the testicular sections are attached to adhesive slides, and the entire procedure is performed directly on the slides (1). With the development of high-throughput sequencing and genome editing technology (19, 38, 39), large amounts of data and samples need to be studied by the method of immunofluorescence. However, the working-on-slide method is inefficient and no longer suitable for high-throughput study. In contrast, slide dropping, tissue loss, sample drying, high staining background, and uneven staining of samples are common problems associated with this method (40, 41). Therefore, an easy-to-use, high-throughput, and high-quality immunofluorescence method should be developed.

In this study, a set of antibodies against zebrafish Ddx4, Piwil1, Sycp3, and Pcna and a high-throughput and high-quality integrated immunofluorescence method for testicular samples were developed. By studying the expression patterns of these proteins in the testes of different fish species, zebrafish, Chinese rare minnow, common carp, blunt snout bream, and rice field eel, an integrated criterion for identifying different types/subtypes of spermatogenic cells was proposed.

## 2 Materials and methods

### 2.1 Fish

One-year-old zebrafish males of the AB strain and one-year-old Chinese rare minnow males were obtained from the China Zebrafish Resource Center, National Aquatic Biological Resource Center (CZRC-NABRC, http://zfish.cn). Two-year-old common carp males, two-year-old blunt snout bream males, two-year-old rice field eel males and 6-month old grass carp were obtained from NABRC, Institute of Hydrobiology, Chinese Academy of Sciences, as previously described (9). The animal experiments were performed under the approval of the Institutional Animal Care and Use Committee of the Institute of Hydrobiology, Chinese Academy of Sciences.

### 2.2 Hematoxylin and eosin (HE) staining

The testes of the selected adult fish were fixed in Bouin’s fixative for 24 h before processing. After standard histological processing, the samples were embedded in paraffin and sectioned at 5 μm for hematoxylin and eosin (HE) staining. The HE staining was conducted using a HE staining kit (Solarbio, Beijing, China) according to the manufacturer’s protocol. The slides were viewed and the photos were taken under an upright microscope with a digital camera (Zeiss).

### 2.3 Generation, purification, and labeling of polyclonal antibodies

**Figure 1:**
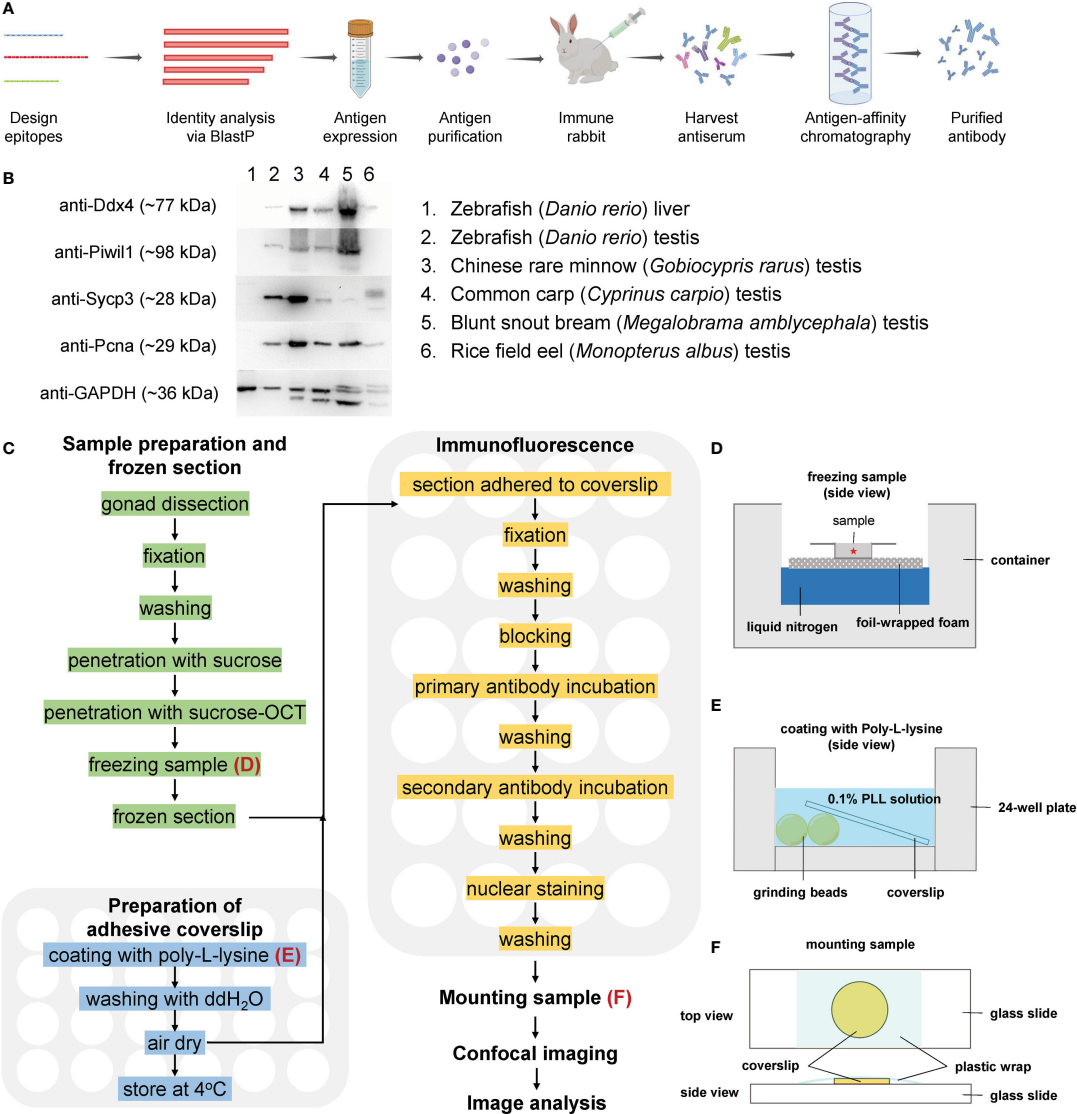
A simplified workflow and validation of antibodies by Western blot. **(A)** A simplified flow chart of antibody preparation. **(B)** Validation of the antibodies by Western blot. **(C)** Route diagram of the experimental procedures; the preparation of adhesive coverslips and immunofluorescence are performed in 24-well plates; **(D)** The schematic showing freezing sample in OCT; **(E)** The schematic showing coating with PLL; **(F)** The schematic showing mounting sample.

A custom antibody production service (Bioyear, China) was utilized to generate rabbit polyclonal antibodies. The epitopes sequences of zebrafish Ddx4, Piwil1, Sycp3, and Pcna are listed in **Table 1**. All antibodies were purified from anti-serum using antigen-affinity chromatography as described previously (**Figure 1A**) (43). Briefly, 10 mg purified antigen protein was coupled to 2 mL cyanogen bromide (CNBr) activated agarose (Sangon Biotech, China). Forty mL of antiserum was purified by 2mL of antigen-coupled activated agarose. For Western blot analysis and single antibody immunofluorescence, the polyclonal antibodies were diluted in 50% glycerol in PBS and stored at -80 °C. For fluorescent labeling, the antibodies were stored in PBS at -80 °C. For immunofluorescence co-staining, the antibodies of Sycp3, Pcna and Ddx4 were conjugated with Alexa Fluor 488, 568 and 680, respectively using Alexa Fluor^®^ Antibody Labeling Kits according to the manufacturer’s manual (Thermo Fisher).

**Table 1:** The epitope sequences of the antibodies Ddx4, Pcna, and Sycp3.

Protein name	Epitope position	Epitope sequence
Ddx4	516–666 amino acids	KRDQLLELLRATGNERTMVFVETKRSADFIATFLCQEKISTTSIHGDREQREREKALSDFRLGHCPVLVATSVAARGLDIEQVQHVVNFDM PSSIDEYVHRIGRTGRCGNTGRAVSFFNPESDTPLARSLVKVLSGAQQVVPKWLEEVAFS
Piwil1	40-54 amino acids	EGQLVGRGRQKPAPG (42)
Sycp3	full length	MAAAGRKQNKKTKHTDGVVTDLKAFDFTVLEEKKESGSDEDTRDDETPIIDKLSKKRSADTFDDELNSGVGNEVQSMLERFGADISK AMQTKRKRLEVLTKNSLKGSTQKLEQMWKTQQNQRQKLTQDHSQQVFSVLQQWESDVLKSEEQEEKLNNLFRQQLKLFQQARVVQK QKIKTINDLHEQFVKNIEEMEKSHEAFLQGTQMELRKEMALLQKKIMMDTQQQEMATVRKSLQSMLF
Pcna	full length	MFEARLVQGSILKKVLEALKDLITEACWDVSSSGISLQSMDSSHVSLVQLTLRSDGFDSYRCDRNLAMGVNLSSMSKILKCAGNEDII TLRAEDNADALALVFETLNQEKVSDYEMKLMDLDVEQLGIPEQEYSCVVKMPSGEFARICRDLSQIGDAVMISCAKDGVKFSASGE LGTGNIKLSQTSNVDKEDEAVTIEMNEPVQLIFALNYLNFFTKATPLSKTVTLSMSADIPLVVEYKIADMGHVKYYLAPKIDEESS

### 2.4 Western blot

Total protein extracted from testis of adult fish was diluted with RIPA lysis buffer (P0013C, Beyotime, China) to a final concentration of 10 mg/mL. Western blot was performed as previously described (44). Briefly, the rabbit anti-Ddx4 antibody, anti-Sycp3 antibody, anti-Pcna antibody, and anti-Piwil1 antibody were diluted for 1:2000 in the TBST consisting of 1% skim milk. The abundance of GAPDH was examined as a loading control using anti-GAPDH mouse monoclonal antibody (AF0006, Beyotime, China) with a dilution ratio 1:4000. Horseradish peroxidase-conjugated anti-rabbit antibody and anti-mouse antibody were used as secondary antibodies with a dilution of 1:3000. The signal was detected with the HRP Substrate, and the image was captured using a Bio-Rad ChemiDocMP System.

### 2.5 High-throughput immunofluorescence and analysis of confocal images

A pipeline of high-throughput immunofluorescence and analysis of confocal images comprises the following steps: (1) preparation of adhesive coverslip, (2) sample preparation and frozen section, (3) immunofluorescence, (4) sample mounting and confocal imaging, and (5) image processing and analysis. A simplified workflow is illustrated in **Figure 1**.

#### 2.5.1 Preparation of adhesive coverslips

To achieve high-throughput immunofluorescence, 24-well plates were used for the following manipulations. To facilitate tissue section adherence, round coverslips with a diameter of 12 mm were coated with Poly-L-lysine (PLL) and evaluated as previously described (45). Briefly, eight zirconia grinding beads were placed in each well; a coverslip was placed on the well, 500 μL PLL solution (1 mg/mL, Sigma-Aldrich) was added to each well (**Figure 1B**) for 30 min. After removing PLL solution, 1 mL ddH_2_O was added to each well to rinse the coverslip for 5 min. Finally, the PLL-coated coverslips were air-dried in an oven at 60°C. The 24-well plate containing adhesive coverslips was sealed with plastic wrap and stored at 4°C.

#### 2.5.2 Sample preparation and frozen sections

Fish were anesthetized with 0.2 mg/mL MS-222 in fish system water, and the testes were dissected. For zebrafish, the testis was fixed in 4% paraformaldehyde (PFA) at room temperature (RT) (~25°C) for two hours. After removing the adipose tissue, the fixed testes were processed for frozen sections according to a previously published method with several modifications (46). Briefly, the fixed testes were washed with pH 7.0 PBST buffer (PBS with 0.1% TritonX-100) 3 times for 5 min each, and then transferred into an ascending series of sucrose in PBS for 30 min each (5%, 8%, 12%, 16%, and 20% sucrose). Samples were washed in 20% sucrose/OCT (optimal cutting temperature compound, Tissue-Tek) (2:1) and 20% sucrose/OCT (1:1) for 30 min each and embedded in 20% sucrose/OCT (1:1) in a frozen mold on foil-wrapped floating foam on liquid nitrogen (**Figure 1C**), and the frozen samples were stored at -80 °C. The embedded testis was cut into sections of 12 μm, and the sections were collected on PLL-coated coverslips.

#### 2.5.3 Immunofluorescence

The sections were air-dried and fixed with 4% PFA at RT for 20 min. The fixed sections were washed with PBST 3 times for 5 min each, permeabilized with PBST for 30 min, and washed with PBSBDT (2% BSA, 1% DMSO, 0.1% Triton X-100 in PBS) 3 times for 5 min each. The sections were then blocked in PBSBDT for one hour at RT. After blocking, the sections were incubated in the diluted antibodies (1:500 in PBSBDT) overnight at 4°C. After washing with PBSBDT 3 times for 5 min each, the sections were incubated in secondary antibody (Goat-anti-Rabbit Alexa Fluor 488, 1 mg/mL in stock, 1:1000 in PBSBDT) (Thermo Fisher) at RT for two hours. The sections were then washed with PBSBDT 3 times for 5 min each, stained with DAPI at 1 μg/mL at RT for 10 min, and washed with PBST 3 times for 5 min each. To test the maximum stringency, 15 min instead of 5 min was used for the washing steps, and no obvious differences were found on the staining results, thus 5 min was generally used in the present study. For the co-staining of different antibodies, the incubation of secondary antibody and the following wash steps were omitted.

#### 2.5.4 Sample mounting and confocal imaging

After immunofluorescence, each section was mounted with 10 μL VECTASHIELD^®^ mounting medium and covered with a piece of plastic wrap (**Figure 1D**). The samples were stored at 4°C in a dark box until imaging under a confocal microscopy. Confocal images were acquired using a laser-scanning confocal inverted microscope (SP8, Leica) with a 20x air objective (numerical aperture 0.75) or a 63x oil-immersed objective (numerical aperture 1.4). For each channel the pin hole was set to 1 Airy unit (AU), that was 0.26 μm for the 20x objective or 0.14 μm for the 63x objective.

#### 2.5.5 Nuclear morphological analysis and cell counting

Morphological criteria of the nucleus such as size (as revealed by the diameter of nucleus), shape, and chromosome condensation of nucleus (as revealed by the relative DAPI intensity), and size and number of nucleoli were used to identify four classes of spermatogonia (SPG) according to the description in **Table 2**. The morphological criteria were also used to distinguish SPG, primary spermatocyte (SPC-I), secondary spermatocyte (SPC-II), spermatid (SPD) and spermatozoa (SPZ), according to a previous report (2). The clay ball model was used to calculate the cell number in a cyst, and this method were only applied to the cysts containing Class 3 and Class 4 SPG (47). This model relies on the counting of cell number at the largest cyst section. To find the largest cyst section in each class of cysts, we reviewed over 200 cyst sections containing Class 3 or Class 4 SPG in 94 sections from four independent testes (at least 20 sections for each testis), the top 9 largest cyst surfaces for Class 3 SPG and the top 8 largest cyst surfaces for Class 4 SPG were counted and analyzed. To calculate the diameter of cell nucleus, the perimeter of the nucleus was measured and divided by circular constant.

**Table 2:** Identification of four classes of SPG in zebrafish using morphological criteria.

Cell types	Nuclear morphology	Mean cell number in the largest cyst section(s)
Class 1/SPG-A(1-cell)	large and poorly condensed nucleus with irregular or round shape	/
Class 2/SPG-A(2,4,8-cell)	large and round nucleus, a large nucleolus	/
Class 3/SPG-B(16,32-cell)	large, round or elongated nucleus with one to three visible nucleoli	14.3 ± 1.5 ^a^
Class 4/SPG-B(64,128,256-cell)	small nucleus, condensed chromosome without obvious nucleolus	54.9 ± 10.7 ^b^

^a, b^ Different superscript letters indicate that the mean values differ significantly (P < 0.05).

#### 2.5.6 Fluorescence intensity analysis

Images were adjusted for brightness and contrast with Fiji software (48). To measure the fluorescent intensity of Ddx4, Piwil1 and DAPI, 16 bit images were acquired. The intensity measurement was performed according to the ImageJ User Guide. The images from the same testicular samples were set into the same threshold. At least three images/regions from three independent testicular samples were measured. Within one image/region, the relative intensity of DAPI, Ddx4 or Piwil1 for each cell type was measured and calculated relative to the intensity of the SPG-A(2,4,8-cell) within the same image/region.

#### 2.5.7 Statistical analysis

**Figure 2:**
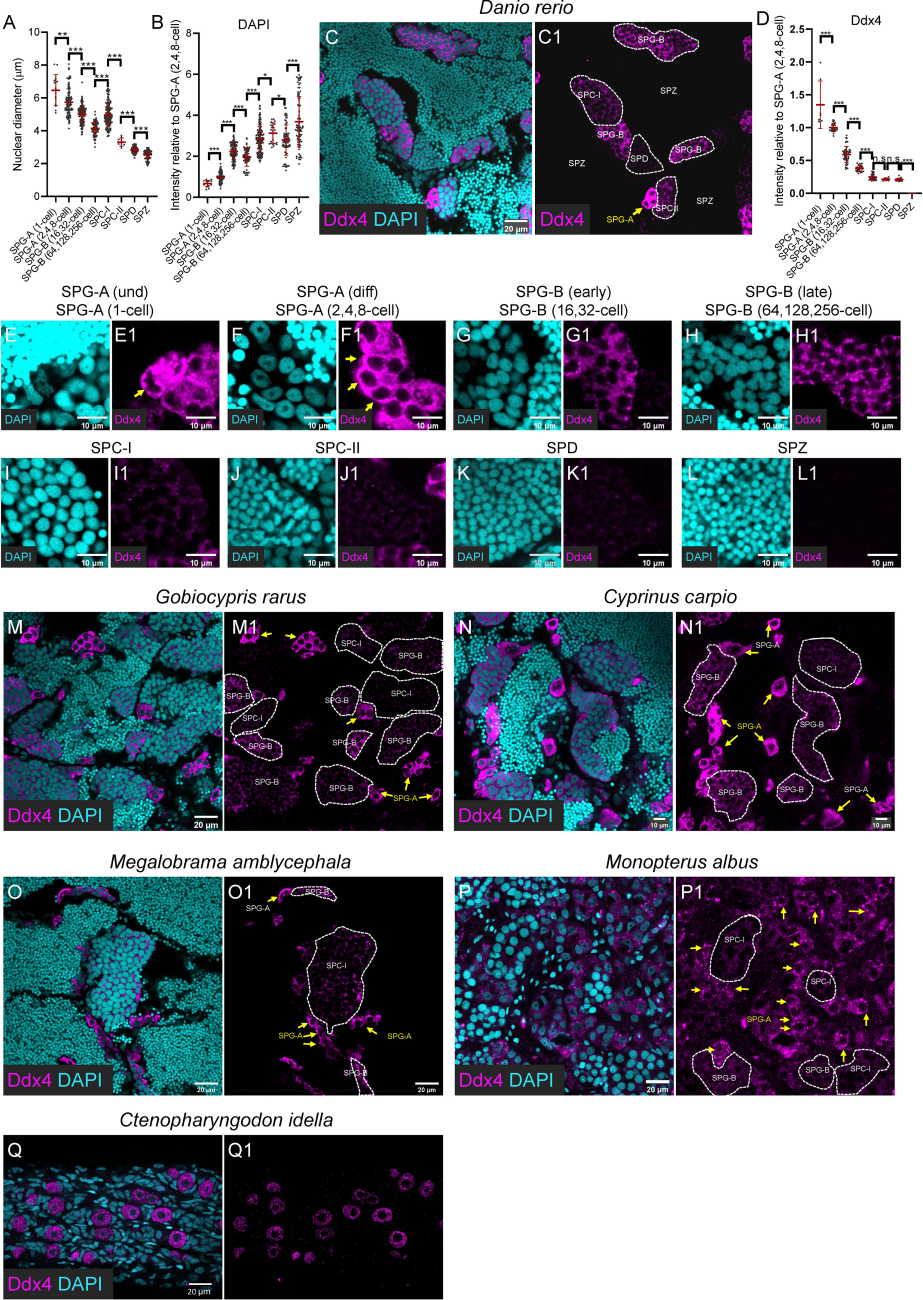
Progressively decreased expression of Ddx4 along spermatogenesis. **(A)** The graph showing the nuclear diameter of different types of spermatogenic cells. **(B)** The graph showing the DAPI intensity of different types of spermatogenic cells relative to that of the SPG-A(2,4,8-cell). **(C, C1)** Representative images of a zebrafish testis section showing the expression of Ddx4 (magenta) co-stained with DAPI (cyan). **(D)** The graph showing the Ddx4 intensity of different types of spermatogenic cells relative to that of the SPG-A(2,4,8-cell). **(E-L, E1-L1)** The Representative images showing the SPG-A(1-cell) **(E, E1)**, the SPG-A(2,4,8-cell) **(F, F1)**, the SPG-B(16,32-cell) **(G, G1)**, the SPG-B(64,128,256-cell) **(H, H1)**, the SPC-I **(I, I1)**, the SPC-II **(J, J1)**, the SPD **(K, K1)** and the SPZ **(L, L1)**. **(M-P, M1, P1)** The representative images showing the expression of Ddx4 in *Gobiocypris rarus* **(M, M1)**, *Cyprinus carpio* **(N, N1)**, *Megalobrama amblycephala* **(O, O1)**, *Monopterus albus* **(P, P1)** and *Ctenopharyngodon idella* **(Q, Q1)**. SPG-As are indicated by the arrows, and other types of spermatogenic cells are circled by dashed lines. All data were collected from three independent testicular samples. All values are the mean ± STD. Unpaired and two-tailed Student’s t-test were used to compare means. *P<0.05; **P<0.01; ***P<0.001, n.s. not significant.

**Figure 3:**
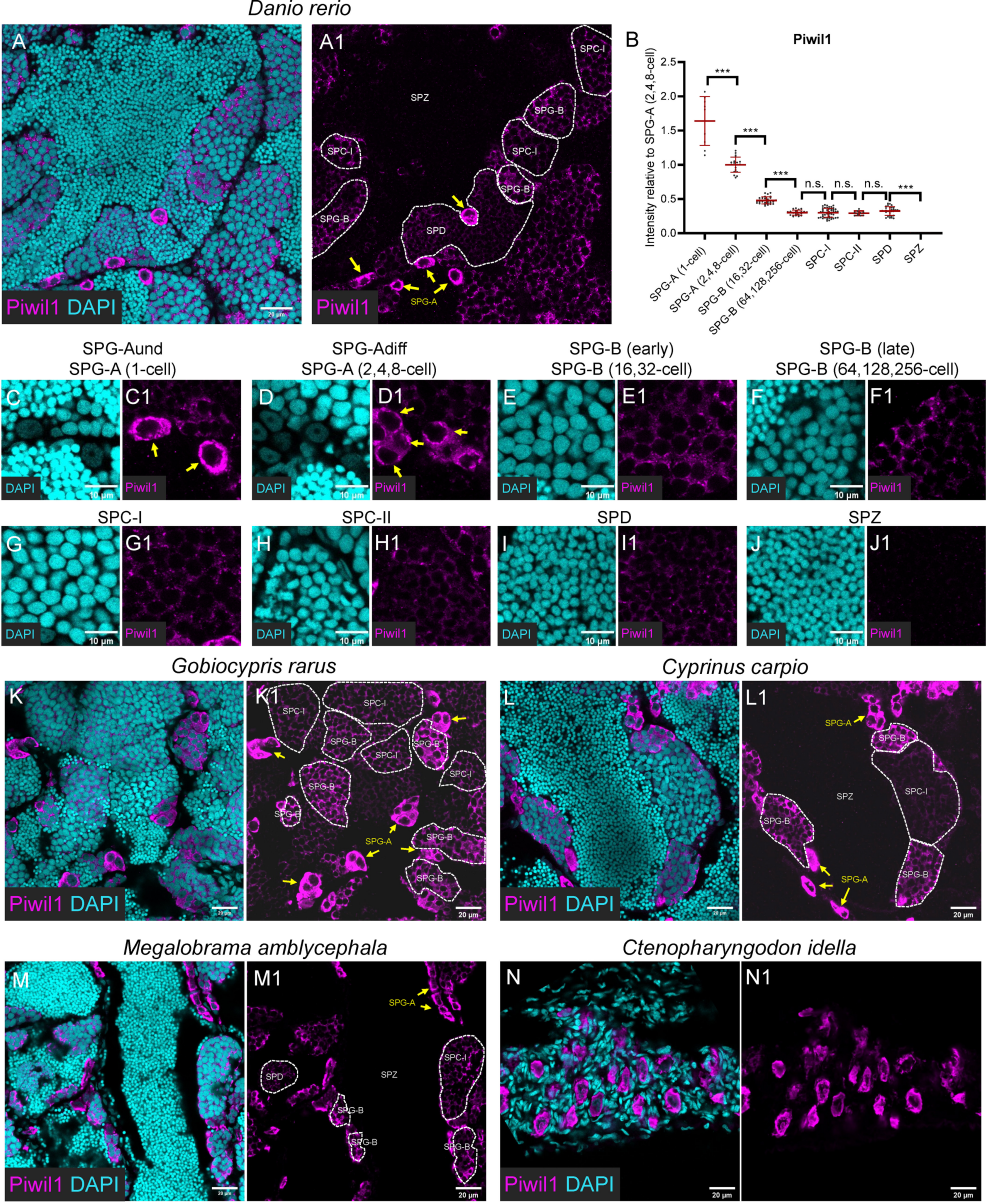
Piwil1 was highly expressed in the SPG-A, and expressed in the SPD but not in the SPZ. **(A, A1)** Representative images of a zebrafish testis section showing the expression of Piwil1 (magenta) co-stained with DAPI (cyan). **(B)** The graph showing the Piwil1 intensity of different types of spermatogenic cells relative to that of the SPG-A(2,4,8-cell). **(C-J, C1-J1)** The Representative images showing the SPG-A(1-cell) **(E, E1)**, the SPG-A (2,4,8-cell) **(F-F1)**, the SPG-B(16,32-cell) **(G, G1)**, the SPG-B(64,128,256-cell) **(H, H1)**, the SPC-I **(I, I1)**, the SPC-II **(J, J1)**, the SPD **(K, K1)** and the SPZ **(L, L1)**. **(K-M, K1-M1)** The representative images showing the expression of Piwil1 in *Gobiocypris rarus* **(K, K1)**, *Cyprinus carpio* **(L, L1)**, *Megalobrama amblycephala* **(M, M1)** and *Ctenopharyngodon idella* **(N, N1)**. SPG-As are indicated by the arrows, and other types of spermatogenic cells are circled by dashed lines. All data were collected from three independent testicular samples. All values are the mean ± STD. Unpaired and two-tailed Student’s t-test were used to compare means. ***P < 0.001, n.s. not significant.

The data in **Figures 2**, **3** were plotted using Graphpad Prism software. The values were presented as mean ± standard deviation (STD) and were analyzed using unpaired and two-tailed Student’s t-test. Statistical significance is represented by asterisks (*P < 0.05, **P < 0.01, ***P < 0.001). The data in **Table 2** were presented as mean ± SEM (standard error of the mean) and analyzed *via* unpaired and two-tailed Student’s t-test. The analysis was performed using the Graphpad Prism software. The statistical significance in different groups was considered to be P < 0.05.

## 3 Results

### 3.1 Validation of antibodies and development of high-throughput immunofluorescence

Antibodies against zebrafish Ddx4, Piwil1, Sycp3, and Pcna were generated against the antigens (**Table 1**), and purified using antigen-affinity chromatography (**Figure 1A**). Corresponding proteins at specific sizes were detected by Western blot with these antibodies using zebrafish testicular samples but not liver sample (negative control, **Figure 1B**), indicating that these antibodies are applicable in zebrafish. An alignment of the sequences for each protein from the selected fish was performed, and each protein showed high homologous identity among different species (**Supplementary Figures 1**-**4**). The cross-species reactivity of the antibodies was further analyzed by Western blot. Immunoblotted bands of around 77 kDa, 28 kDa, and 29 kDa were detected using the anti-Ddx4, anti-Sycp3, and anti-Pcna antibodies, respectively, in all the test fish species (**Figure 1B**), indicating that the raised anti-Ddx4, anti-Sycp3, and anti-Pcna antibodies could be used to detect corresponding proteins by Western blot in these fish species. Regarding the anti-Piwil1 antibody, predicated Piwil1 protein with a size of 98 kDa could be detected in all the other fishes except rice field eel (**Figure 1B**).

To obtain a general overview of the spermatogenic status of these fishes at sampling, we first conducted paraffin-based section and HE staining. Although we could identify SPG, SPC-I, and SPD with typical characteristics based on the morphological characters according to a previous report (28) (**Supplementary Figure 5**), it is difficult and extremely time consuming for us to identify different subtypes of these spermatogenic cells. Then we aimed to develop a high-throughput immunofluorescence method using 24-well plate format based on immunofluorescence analyses of testicular samples with these antibodies (**Figures 1C–F**). Briefly, different testicular samples were collected, frozen in liquid nitrogen (**Figure 1D**), and stored at –80°C. After frozen sectioning, the sections were attached to the PLL-treated coverslips (**Figure 1E**), and immunofluorescence was performed in 24-well plates as described in **Figures 1C-F**. A specialist could operate two or more plates of samples, i.e., more than 48 samples, at the same time without worrying about slide dropping, tissue loss, sample drying, high staining background, or uneven staining. The entire process from sample preparation to confocal imaging generally takes two days. By contrast, with the classical working-on-slide immunofluorescence method, it will be a tremendous amount of work to perform immunofluorescence on so many section samples. At times, the staining was uneven with higher signals at the marginal regions (**Supplementary Figure 6**).

### 32 Identification of the eight major types of spermatogenic cell based on morphological criteria

SPC-I, SPC-II, SPD and SPZ were easily identified based on nuclear morphology according to a previous report (2). SPC-I was characterized by its large nucleus, condensed chromosomes, and high frequency of visibility which reflects the long duration of meiosis prophase I (2). SPC-II was generally adjacent to the SPC-I and SPD, and some of them were undergoing cell division (**Figures 2J**, **3H**). SPD and SPZ were both present in large numbers in a cyst and were usually adjacent to each other, with SPD having larger and fewer condensed nuclei than SPZ.

As to the SPG population, they were first divided into four classes based on nuclear morphology (**Table 2**). Class 1 SPG was single-located, containing a large nucleus with poorly condensed chromatin (diameter: 6.47 ± 0.95 μm; relative DAPI intensity: 0.67 ± 0.16) of irregular or round shape, which could be identified as SPG-A(1-cell) (previously named as SPG-A(und)) (**Figure 2E**, **Figure 3C**). Class 2 had round and large nucleus (diameter: 5.74 ± 0.82; relative DAPI intensity: 1 ± 0.24), and usually with a large nucleolus, which could be easily identified as SPG-A(2,4,8-cell) (previously named as SPG-A(diff)) (**Figures 2F**, **3D**). Class 3 had relatively large (diameter: 5.01 ± 0.53; relative DAPI intensity: 2.24 ± 0.39), round or elongated nucleus with one to three visible nucleoli (**Figures 2G**, **3E**). Class 4 had relatively small nucleus and condensed chromatin (diameter: 4.14 ± 0.40; relative DAPI intensity: 1.95 ± 0.44) without obvious nucleolus (**Figures 2H**, **3F**). Both Class 3 and Class 4 SPG could be identified as SPG-B. To determine which one is at earlier generations, we counted the cell number in the largest cyst section and calculated the number in the cyst according to the clay ball model (47). The number for the Class 3 cysts was 14.3 ± 1.5, indicating the cell number in the largest Class 3 cysts was 32. Therefore, we assumed that Class 3 cysts should contain either 16 or 32 SPG-B cells, although we could not exactly figure out which cyst contained 16 cells. For Class 4 cysts, the number was 54.9 ± 10.7, indicating cell number in the largest Class 4 cysts was 256. Therefore, we assumed that Class 4 cysts should contain 64, 128 or 256 cells, although we could not exactly figure out which cyst contained 64 or 128 SPG-B cells in our study (**Table 2**). Finally, based on the cell number in the largest cysts of each class, we named Class 3 SPG as SPG-B(16,32-cell) (previously named as SPG-B(early)), and Class 4 SPG as SPG-B(64,128,256-cell) (previously named as SPG-B(late)) (**Table 2**).

Finally, based on morphological criteria, including cell number in the largest cyst sections, nuclear size (**Figure 2A**), nuclear shape, chromosome condensation (indicated by relative DAPI intensity, **Figure 2B**), and nucleolus number, eight major types of spermatogenic cells were identified (**Figures 2A, B**, **Table 2**). We then focused on the expression pattern of Ddx4, Piwil1, Sycp3, and Pcna in each type of spermatogenic cell.

### 3.3 Progressively decreased expression of Ddx4 along spermatogenesis

As shown by immunofluorescence against anti-Ddx4 antibody, Ddx4 was highly expressed in SPG-A(1-cell) and SPG-A(2,4,8-cell) of zebrafish (**Figure 2**, arrow in **C, C1; D; E, E1; F, F1**). The expression level became significantly lower in SPG-B, including SPG-B(16,32-cell) and SPG-B(64,128,256-cell) (**Figures 2D, G, G1, H, H1**). As SPG-B(64,128,256-cell) differentiated into SPC-I, the expression level of Ddx4 was dramatically reduced (**Figures 2D, I, I1**). Both SPC-II and SPD showed weak expression of Ddx4, while SPZ showed no expression of Ddx4 (**Figures 2D, J, J1, K, K1, L, L1**).

Different types of spermatogenic cell were visualized by the anti-Ddx4 antibody in the testicular samples of Chinese rare minnow, common carp, blunt snout bream, rice-field eel and grass carp. All these fishes showed similar expression patterns of Ddx4 as zebrafish (**Figures 2M-Q, M1-Q1**). Notably, in the testis of rice field eel, expression of Ddx4 could be seen in the SPG-A, but not in the SPG-B or SPC-I, suggesting that Ddx4 could be used for identifying SPG-A in rice field eel (**Figures 2P, P1**; arrows indicate SPG-As). In the 6-month grass carp, there were only singly-located germ cells with strong Ddx4 staining signals in the gonad (**Figures 2Q, Q1**), indicating those germ cells were undifferentiated germline stem and progenitor cells (GSPCs) (18). In summary, the expression level of Ddx4 decreases as spermatogenesis progresses in fishes.

### 3.4 High expression of Piwil1 in SPG-A

In zebrafish testis, the anti-Piwil1 signals were notably detected in SPG-A cells with significant high levels (**Figures 3A, A1, B**). In details, Piwil1 showed extremely high expression level in the entire cytoplasm region of SPG-A(1-cell) (**Figures 3C-C1**), and its expression in the perinuclear germ granules was high in SPG-A (**Figures 3C, C1, D, D1**). The average expression of Piwil1 became significantly low in SPG-B(16,32-cell) (**Figures 3B, E, E1**), decreased to a basal level in SPG-B(64,128,256-cell) (**Figures 3B, F, F1**), SPC-I (**Figure 3B, G, G1**), SPC-II (**Figures 3H, H1**), and SPD (**Figures 3I, I1**), and was not detectable in SPZ (**Figures 3J-J1**).

The anti-Piwil1 antibody could be used to detect spermatogenic cells of Chinese rare minnow, common carp, blunt snout bream, and grass carp, but not rice field eel (**Figures 3K, K1, L, L1, M, M1**), which was consistent to the results of Western blot (**Figure 1B**). Like what was observed in zebrafish, Piwil1 also showed high expression level in SPG-A in Chinese rare minnow, common carp, blunt snout bream (**Figures 3K, K1, L, L1, M, M1**), and in GSPCs of grass carp (**Figures 3N, N1**).

### 35 Characterization of different subtypes of SPC-I by co-staining of Sycp3 and Pcna

**Figure 4:**
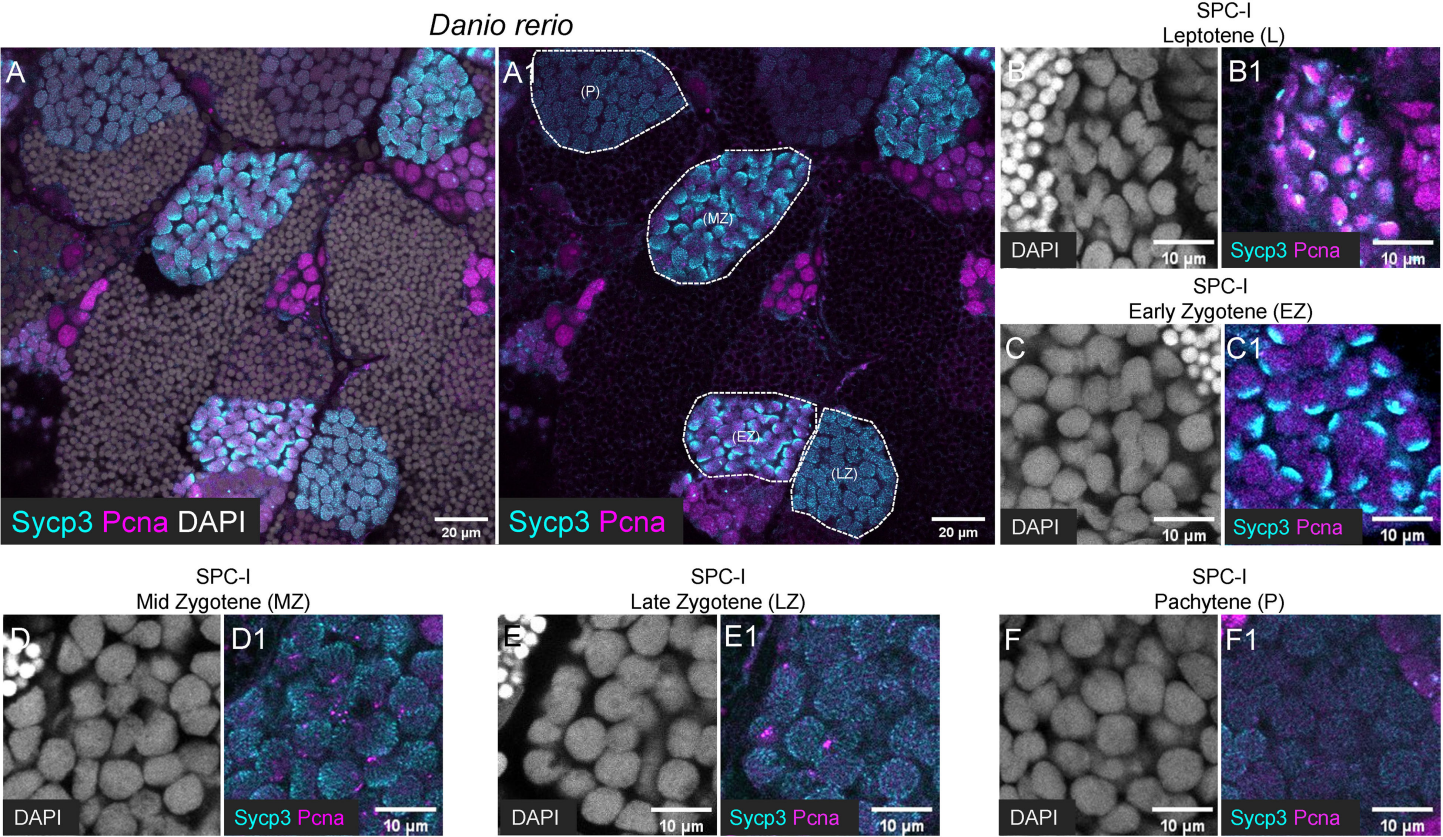
Characterization of different subtypes of SPC-I by co-staining of Sycp3 and Pcna. **(A, A1)** The representative images of a zebrafish testis section showing the co-staining of Sycp3 (cyan), Pcna (magenta) and DAPI (grey). **(B-F, B1-F1)** The representative image showing the SPC-I (Leptotene/L) **(B, B1)**, the SPC-I (Early Zygotene/EZ) **(C, C1)**, the SPC-I (Mid Zytotene/MZ) **(D, D1)**, the SPC-I (Late Zygotene/LZ) **(E, E1)** and the SPC-I (Pchytene/P) **(F, F1)**. Different subtypes of SPC-I are circled by dashed lines.

In zebrafish testis, we found that Sycp3 have distinct expression patterns in different subtypes of SPC-I (**Figures 4A, A1**). In detail, Sycp3 started to show polar expression in one side of the nucleus of SPC-I at leptotene stage (SPC-I Leptotene) (**Figures 4B, B1**), and the polar expression became more obvious in SPC-I at early zygotene stage (SPC-I early-Zygotene) (**Figures 4C, C1**). In SPC-I at mid-zygotene, late-zygotene and pachytene stages, however, the Sycp3 signals expanded to the other side of the nucleus as stripe pattern until occupying the whole nucleus (**Figures 4D, D1, E, E1, F, F1**).

By co-staining of Pcna with Sycp3, a dynamic expression pattern of Pcna during meiosis was observed. In SPC-I Leptotene, Pcna was polarly expressed at the same side as Sycp3 in the nucleus (**Figure 4B1**), while in SPC-I Early-Zygotene, Pcna exhibited a complementary expression pattern to Sycp3 in the nucleus (**Figure 4C-C1**). In SPC-I Mid-Zygotene and SCP-I Late-Zygotene, Pcna was detected as punctuated signals and showed decreased expression level along meiosis (**Figures 4D, D1, E, E1**), and in SPC-I Pachytene, Pcna signals were almost undetectable (**Figure 4F-F1**).

**Figure 5:**
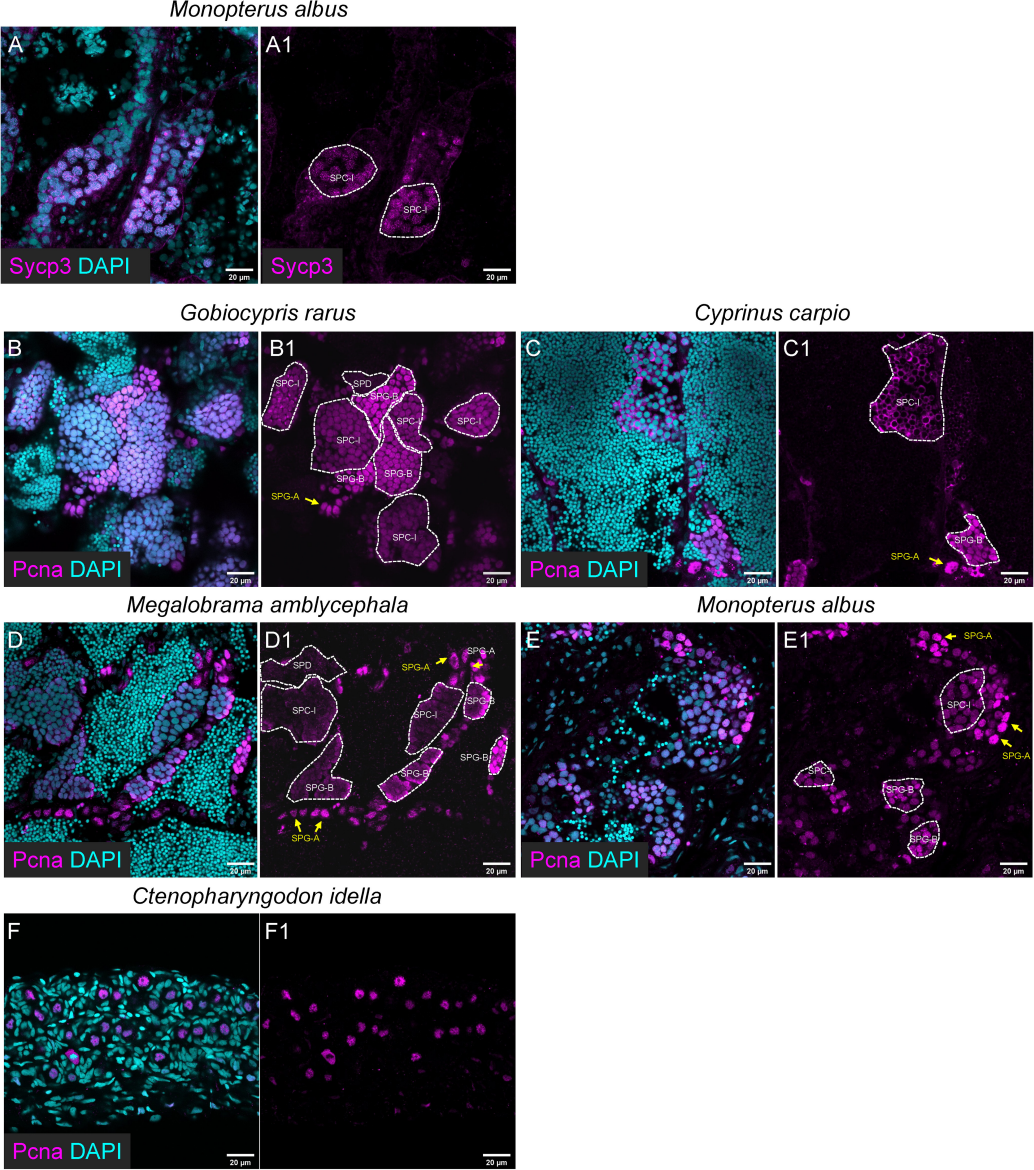
The applicability of the Sycp3 and Pcna antibody for immunofluorescence in the other five selected fishes. **(A, A1)** The representative images showing the expression of Sycp3 in *Monopterus albus*. **(B-E, B1-E1)** The representative images showing the expression of Pcna in *Gobiocypris rarus* **(B, B1)**, *Cyprinus carpio* **(C, C1)**, *Megalobrama amblycephala* **(D, D1)**, *Monopterus albus* **(E, E1)** and *Ctenopharyngodon idella* **(F, F1)**. SPG-As are indicated by the arrows, and other types of spermatogenic cells are circled by dashed lines.

In the other five fish species, the anti-Sycp3 antibody could be only used to detect meiotic cells in rice field eel (**Figures 5A-A1**), and the anti-Pcna antibody could be used to detect mitotic SPG or GSPC and SPC-I in all the five fish species (**Figures 5A-F1**).

### 36 Characterization of different types/subtypes of spermatogenic cells by triple-staining of Ddx4, Sycp3 and Pcna

**Figure 6:**
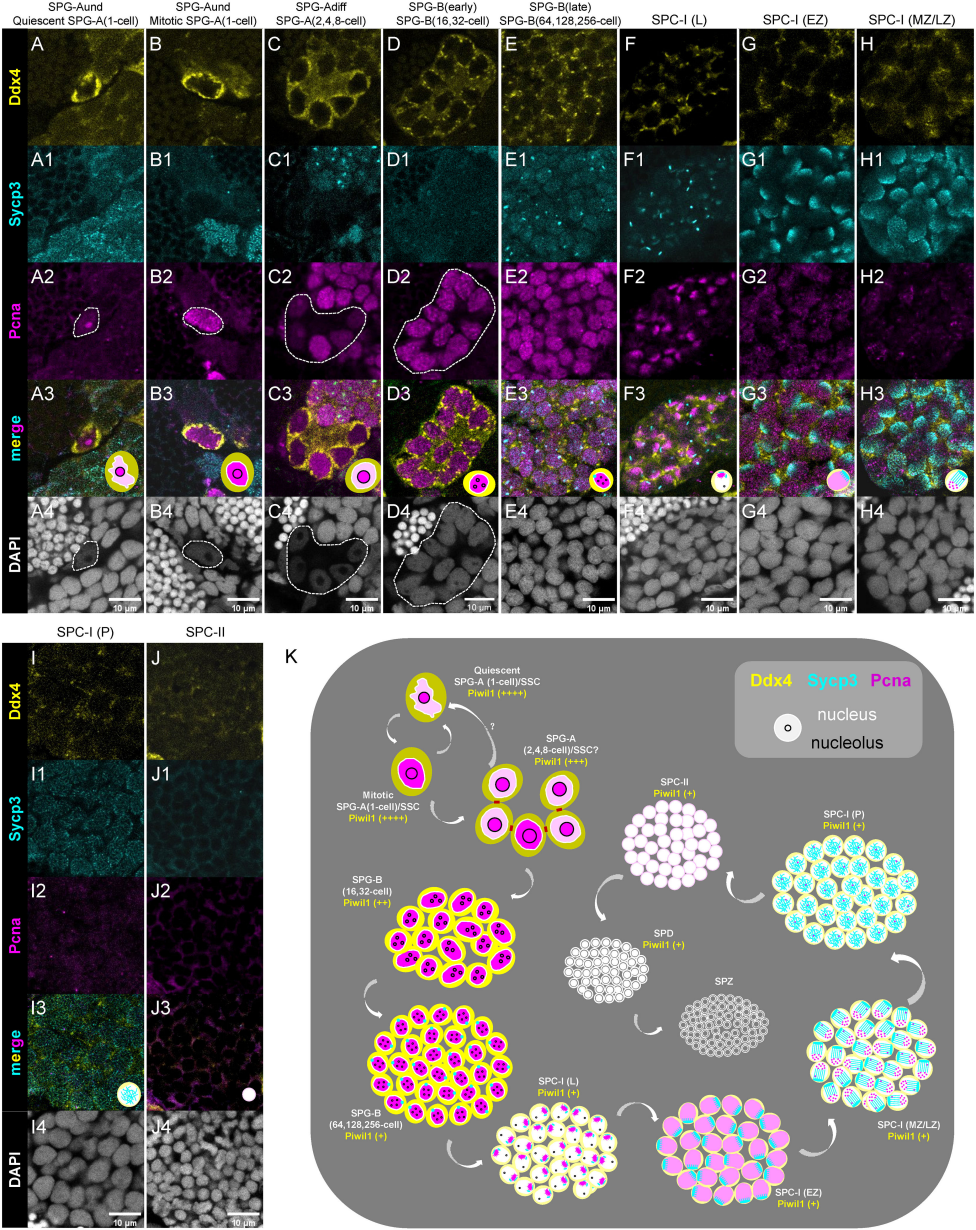
Characterization of different types/subtypes of spermatogenic cells by triple-staining of Ddx4, Sycp3 and Pcna. **(A-J, A1-J1, A2-J2, A3-J3, A4-J4)** The representative images of showing the co-staining of Ddx4 (yellow), Sycp3 (cyan), Pcna (magenta) and DAPI (grey) in the quiescent SPG-A(1-cell) **(A-A4)**, the miotic SPG-A(1-cell) **(B-B4)**, the SPG-A(2,4,8-cell) **(C-C4)**, the SPG-B(16,32-cell) **(D-D4)**, the SPG-B(64,128,256-cell), the SPC-I (L) **(F-F4)**, the SPC-I (EZ) **(G-G4)**, SPC-I (MZ/LZ) **(H-H4)**, SPC-I (P) **(I-I4)**, and the SPC-II **(J-J4)**. Some of the indicated cells are circled by dashed lines to help identifying. **(K)** A schematic diagram showing the expression of the four proteins in the different types and subtypes of spermatogenic cells. SPG-A(1-cell) can be identified by the highest expression of Ddx4 and Piwil1. SPG-Bs including SPG-B(16,32-cell) and SPG-B(64,128,256-cell) are identified by the second highest expression of Ddx4 and the high expression of Pcna, while the later shows nuclear signal of Sycp3. Different subtypes of SPC-I can be identified by the expression patterns of Sycp3 and Pcna. SPD can be distinguished from SPZ by the expression of Piwil1 or Ddx4.

We performed triple-staining of Ddx4, Sycp3 and Pcna to characterize different spermatogenic cells. The expression of Pcna is used to indicate cells with activities of mitosis or DNA repair (49, 50). The subtypes of SPGs were identified based on the criteria of nuclear morphology and Ddx4 expression level (**Figures 6A, E4**), and the subtypes of SPC-I were identified based on the dynamic expression pattern of Sycp3 and Pcna, as described above (**Figures 6F, I4**).

As to the expression of Pcna in SPG cells, some SPG-A(1-cell) cells showed low level of Pcna expression in nucleus (**Figures 6A, A4**), while others showed high level of expression (**Figures 6B, B4**), suggesting that the former SPG-A(1-cell) cells were at quiescent state and the later ones were at mitotic cycle. In SPG-A(2,4,8-cell) cells, Pcna was usually strongly expressed in one or two cells (**Figures 6C, C4**), suggesting that there could be different mitotic progress in a single SPG-A(2,4,8-cell) cyst. Subsequently, Pcna was strongly expressed in all SPG-B cells, consistent with their active proliferation status (**Figures 6D, D4, E, E4**). In some SPG-B(64,128,256-cell) cells, interestingly, Sycp3 started to be expressed as aggregated dots in the nucleus (**Figure 6E1**), indicating that part of SPG-B(64,128,256-cell) cells are accumulating Sycp3 proteins for entering meiosis. In different subtypes of SPC-I cells, low expression of Ddx4 and a dynamic expression pattern of Sycp3 and Pcna were observed (**Figures 6F, I4**). In SPC-II cells, the expression of Ddx4, Sycp3 and Pcna was extremely low (**Figures 6J, J4**).

Finally, we proposed new criteria for the identification of different spermatogenic cells based on the expression level and expression pattern of Ddx4, Piwil1, Sycp3 and Pcna as well as nuclear morphology (**Figure 6K**).

## 4 Discussion

In this study, we generated a set of specific antibodies against zebrafish Ddx4, Piwil1, Sycp3 and Pcna, and used these antibodies to develop a high-throughput immunofluorescence method to identify different spermatogenic cells in different fish species. Based on the expression levels and profiles of Ddx4, Piwil1, Sycp3 and Pcna in different spermatogenic cells in zebrafish, we proposed an immunofluorescence-based criterion for identification of different types of SPG-A, SPG-B, SPC-I, SPC-II, SPD, and SPZ.

We have improved the immunofluorescence method for fish testicular samples in the following ways. 1) To obtain user-friendly and high-throughput results, we utilized 24-well plates for the entire procedure; this allows the experiment to be performed with an automatic pipetting station. 2) Confocal microscopy enables thick sections (10–100 μm) and image analysis at high resolutions. 3) To obtain double or triple fluorescence staining, different rabbit polyclonal antibodies were labeled with different fluorescent colors. 4) To facilitate high-throughput operation, the preparation and storage of adhesive coverslips were optimized. 5) To better maintain cell morphology during freezing, 50% OCT - 10% sucrose instead of 100% OCT was used as the embedding medium. 6) To avoid the use of isopentane (51), a toxic volatile chemical, the samples were frozen on a foil-wrapped foam in liquid nitrogen. Nevertheless, it still needs time to promote this method in most labs involved in fish reproduction or germ cell biology. This method depends heavily on confocal microscopy, which has become a standard instrument for core imaging facilities.

In zebrafish testes, SPG-A and SPG-B both showed high expression of Ddx4 and Piwil1, two factors playing an interactive role in piRNA biogenesis (31), suggesting that piRNA biogenesis is active in SPG. It is worth noting that Pcna was most highly expressed in SPG-B cells undergoing rapid mitotic cell cycle (49, 50), and showed polar localization in the nucleus of SPC-I Leptotene, very close to the localization of Sycp3. It is known that the chromosome axis protein, Sycp3, aggregates and accumulates to one pole of the nucleus during the synapsis initiation (52–54). The polarization of Pcna to one side of the leptotene nucleus could be explained by its role in DNA repair, since meiotic double-strand breaks occur on one side of the nucleus near the telomere bouquet, which also appears at one pole of the nucleus at the leptotene stage (52–57).

In vertebrates, SPG-A(1-cell) is considered as the SSC pool (58). However, in teleosts, whether the stemness of the SPG-A(2,4,8-cell) is flexible under different contexts is still debatable because that, unlike mammals, live-imaging and pulse-labeling studies are lacking in model teleost such as zebrafish (2). In mice, the interconnected syncytia of two to 16 spermatogonia are considered as SSCs in a context-dependent manner, and they share similar nuclear morphology and molecular markers (59). In our study, the nuclear morphology of SPG-A(2,4,8-cell) and the expression pattern of the four proteins in it are also similar to those of SPG-A(1-cell), suggesting that SPG-A(2,4,8-cell) are either SSCs or can achieve stemness under certain condition (**Figure 6K**), which is consistent with a recent single cell sequencing study (60). Nevertheless, more solid conclusion should be concluded by SSC transplantation approach in the future.

All the antibodies raised against zebrafish proteins were tested for their applicability in other fish species including Chinese rare minnow, common carp, blunt snout bream, rice field eel and grass carp. In general, the results of Western blot and immunofluorescence were consistent. For example, the anti-Piwil1 antibody failed to detect the Piwil1 homolog in the rice field eel as shown by Western blot, and it was not applicable to immunofluorescence against rice field eel samples. In contrast, the anti-Sycp3 antibody could visualize specific bands in all the other fishes by Western blot, whereas it only gave specific immunofluorescence signals in the rice field eel samples, indicating the independence on the applicability of the antibodies in different applications (61).

Although the nuclear diameter of a particular subtype of spermatogenic cell was smaller in this study than in the previous study, the relative diameters of the different types of spermatogenic cells were consistent in both studies (21). Both the previous study and our study showed that the nuclear diameter decreased as mitotic division progressed, then increased during meiotic prophase I, and decreased again after the first and second meiotic divisions. The difference in cell diameters between our study and the previous study could be due to different methods of sample processing (27, 62).

In conclusion, we have developed a set of antibodies against zebrafish spermatogenesis-related proteins and utilized them to develop a high-throughput and high-quality immunofluorescence method for fish testicular samples. The antibody set together with the integrated immunofluorescence method provides a simple, practical, and efficient tool to study spermatogenesis in various fishes. One challenge is to obtain high quality images for identification of nuclear morphology of thick samples, which needs to be achieved with advances of microscopic imaging and sample preparation, such as clearing technique.

## Data availability statement

The original contributions presented in the study are included in the article/**Supplementary Material**. Further inquiries can be directed to the corresponding author.

## Ethics statement

The animal study was reviewed and approved by the Institutional Animal Care and Use Committee of the Institute of Hydrobiology, Chinese Academy of Sciences.

## Author contributions

DY: Project administration, Confocal microscopy, Writing-original draft, Funding acquisition. TL: Investigation, Methodology. YL, YW, WH, ZZ: Resources. YS: Supervision, Writing- review and editing, Funding acquisition. All authors contributed to the article and approved the submitted version.
