# Supplementary material for: Identification of fish spermatogenic cells through high-throughput immunofluorescence against testis with an antibody set
Source: Front Endocrinol (Lausanne). 2023 Apr 3;14:1044318. doi: 10.3389/fendo.2023.1044318 (PMC10106697; doi:10.3389/fendo.2023.1044318)
Supplement: Supplementary file 1 [file DataSheet_1.docx]

**Supplementary Figures 1-6**

**
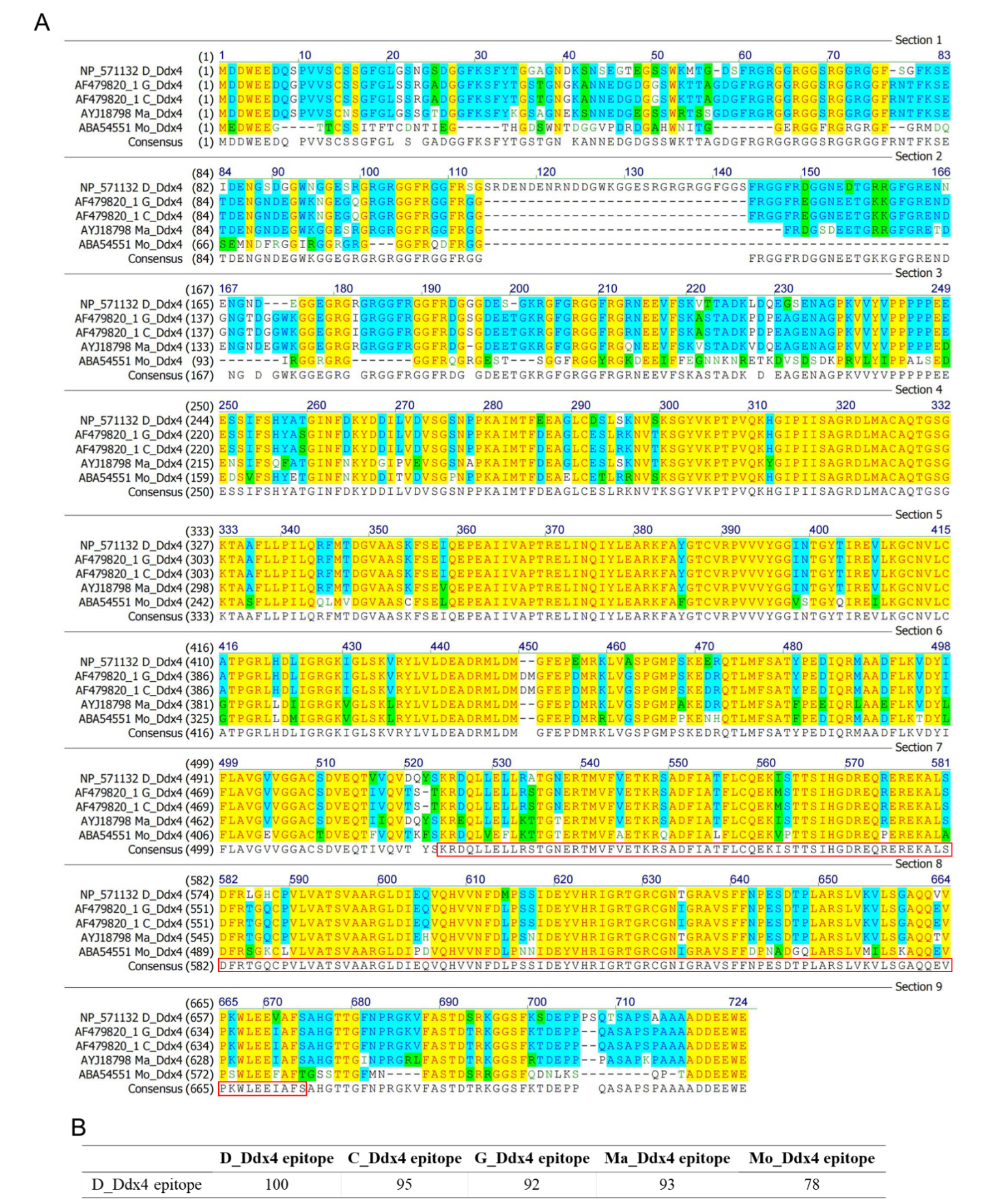
**

**Supplementary Figure 1.**

1. The protein alignment on Ddx4 of different fish species. The GenBank accession numbers is NP_571132 for D_Ddx4, zebrafish (*Danio rerio*) Ddx4, AFA45124 for G_Ddx4, Chinese rare minnow (*Gobiocypris rarus*) Ddx4, AF479820 for C_Ddx4, common carp (*Cyprinus carpio*) Ddx4, AYJ18798 for Ma_Ddx4, AYJ18798 for blunt snout bream (*Megalobrama amblycephala*) Ddx4, ABA54551 for Mo_Ddx4, rice field eel *(Monopterus albus*) Ddx4. The epitope sequence is indicated by the red box. (B) Identity table of Ddx4 epitope.


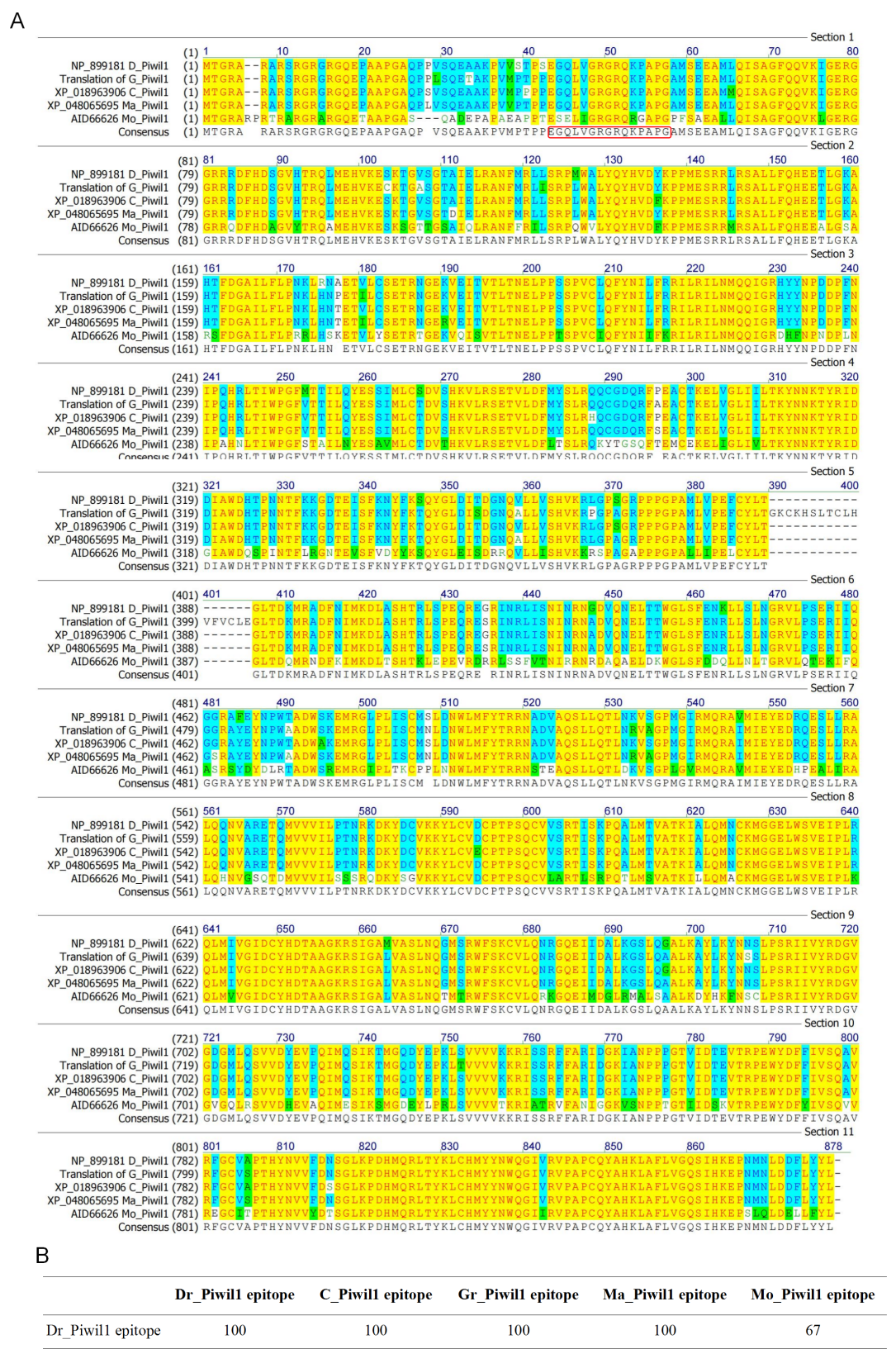


**Supplementary Figure 2.**

1. The protein alignment on Piwil1 of different fish species. The Piwil1 of *Gobiocypris rarus* is absent in the database, and it is predicted from its genome sequence (1). The GenBank accession numbers is NP_899181 for D_Piwil1, zebrafish (*Danio rerio*) Piwil1, XP_018963906 for C_Piwil1, common carp (*Cyprinus carpio*) Piwil1, AYJ18798 for Ma_Piwil1, XP_048065695 for blunt snout bream (*Megalobrama amblycephala*) Piwil1, AID66626 for Mo_Piwil1, rice field eel *(Monopterus albus*) Piwil1. The epitope sequence is indicated by the red box. (B) Identity table of Piwil1 epitope.

**
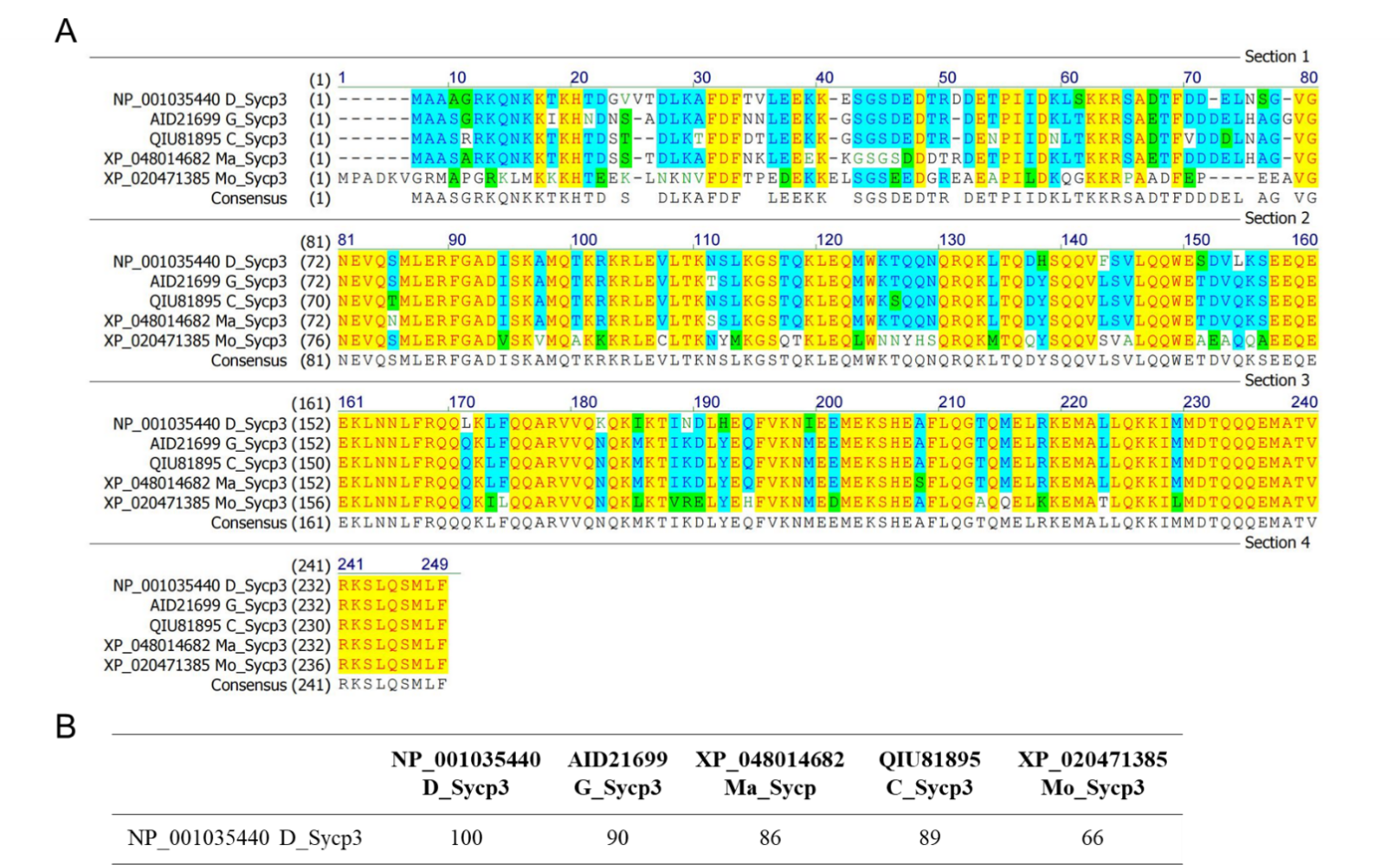
**

**Supplementary Figure 3.**

The protein alignment on Sycp3 of different fish species. The GenBank accession number of each protein is given in front of the protein names. D_Sycp3, zebrafish (*Danio rerio*) Sycp3; G_Sycp3, Chinese rare minnow (*Gobiocypris rarus*) Sycp3; C_Sycp3, common carp (*Cyprinus carpio*) Sycp3; Ma_Sycp3, blunt snout bream (*Megalobrama amblycephala*) Sycp3; Mo_Sycp3, rice field eel *(Monopterus albus*) Sycp3. (B) Identity table of Sycp3 epitope (Full-length sequence).

**
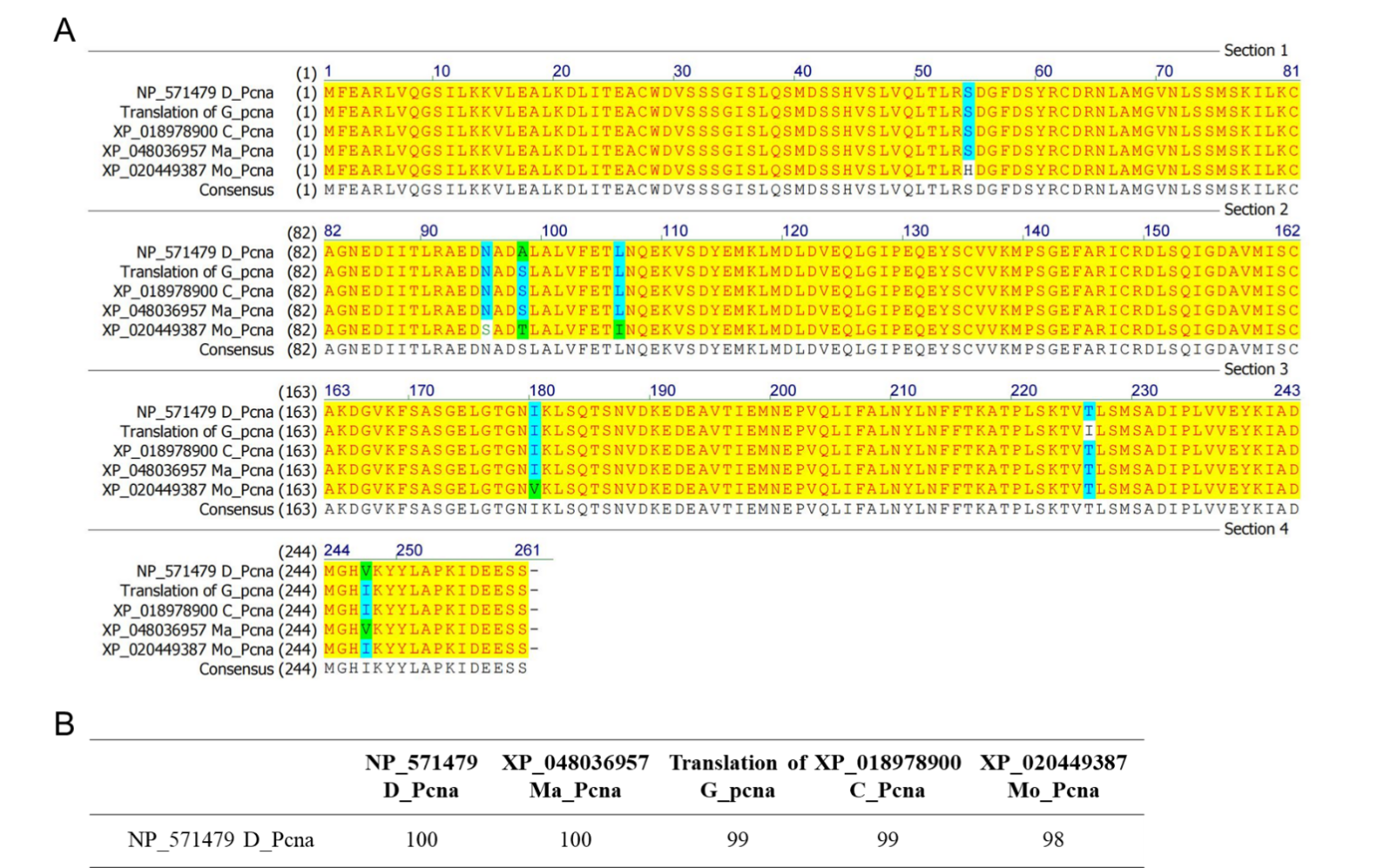
**

**Supplementary Figure 4.**

1. The protein alignment on Pcna of different fish species. The GenBank accession number of each protein is given in front of the protein names. The Pcna of *Gobiocypris rarus* is absent in the database, and it is predicted from its genome sequence (1). D_Pcna, zebrafish (*Danio rerio*) Pcna; G_Pcna, Chinese rare minnow (*Gobiocypris rarus*) Pcna; C_Pcna, common carp (*Cyprinus carpio*) Pcna; Ma_Piwil1, blunt snout bream (*Megalobrama amblycephala*) Pcna; Mo_Pcna, rice field eel *(Monopterus albus*) Pcna. (B) Identity table of Pcna epitope (Full-length sequence).


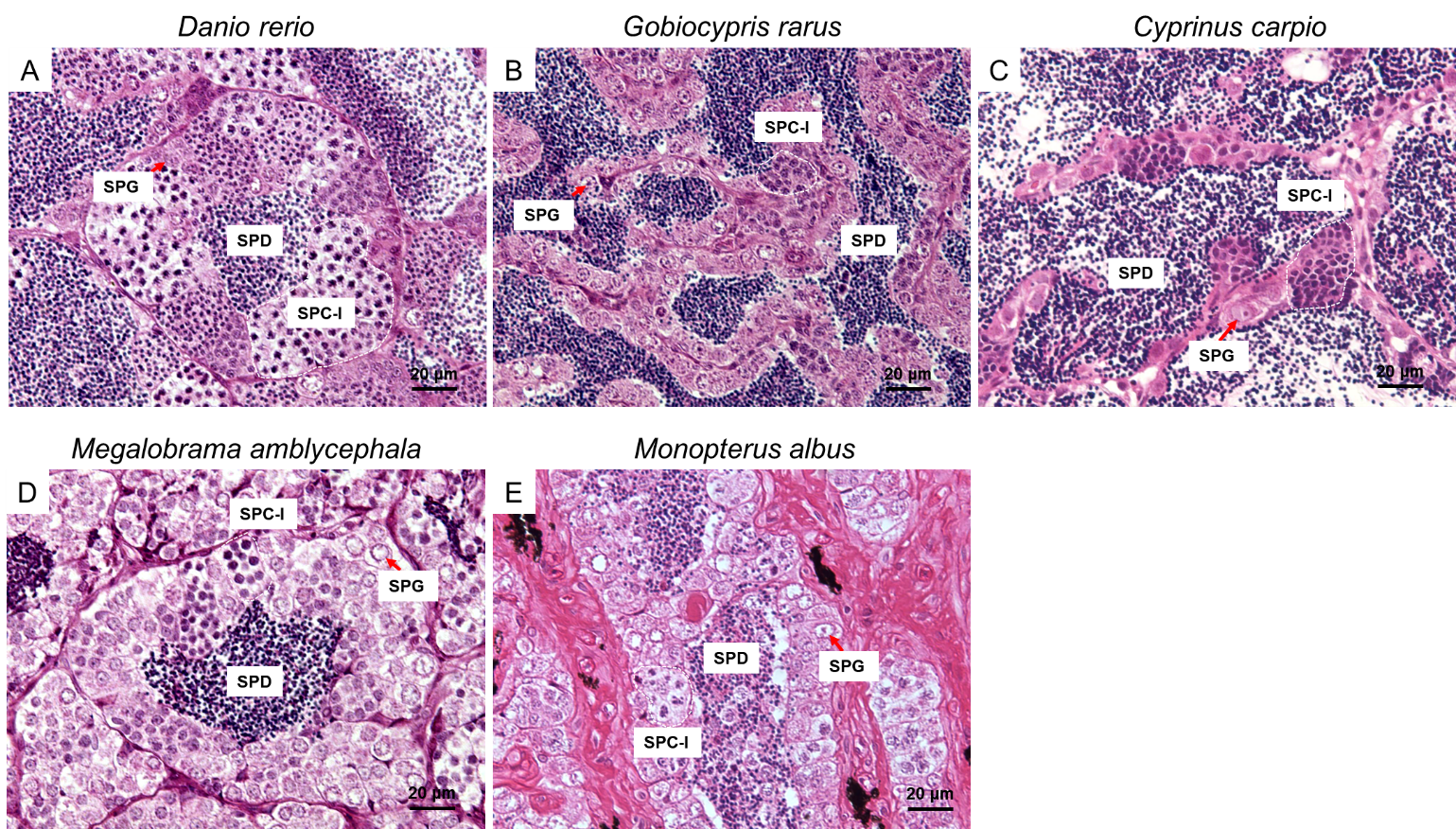


**Supplementary Figure 5.**

The hematoxylin-eosin staining on the matured testes of zebrafish (*Danio rerio*) (A), Chinese rare minnow (*Gobiocypris rarus*) (B), common carp (*Cyprinus carpio*) (C), blunt snout bream (*Megalobrama amblycephala*) (D), and rice field eel (*Monopterus albus*) (E). It is difficult to tell the identity of each cell on the sections based on morphology, so that only the representative SPGs (red arrows), SPC-I (outlined with white dash lines) and SPZs are labeled. SPG, spermatogonia; SPC-I, primary spermatocyte; SPZ, spermatozoa.

**
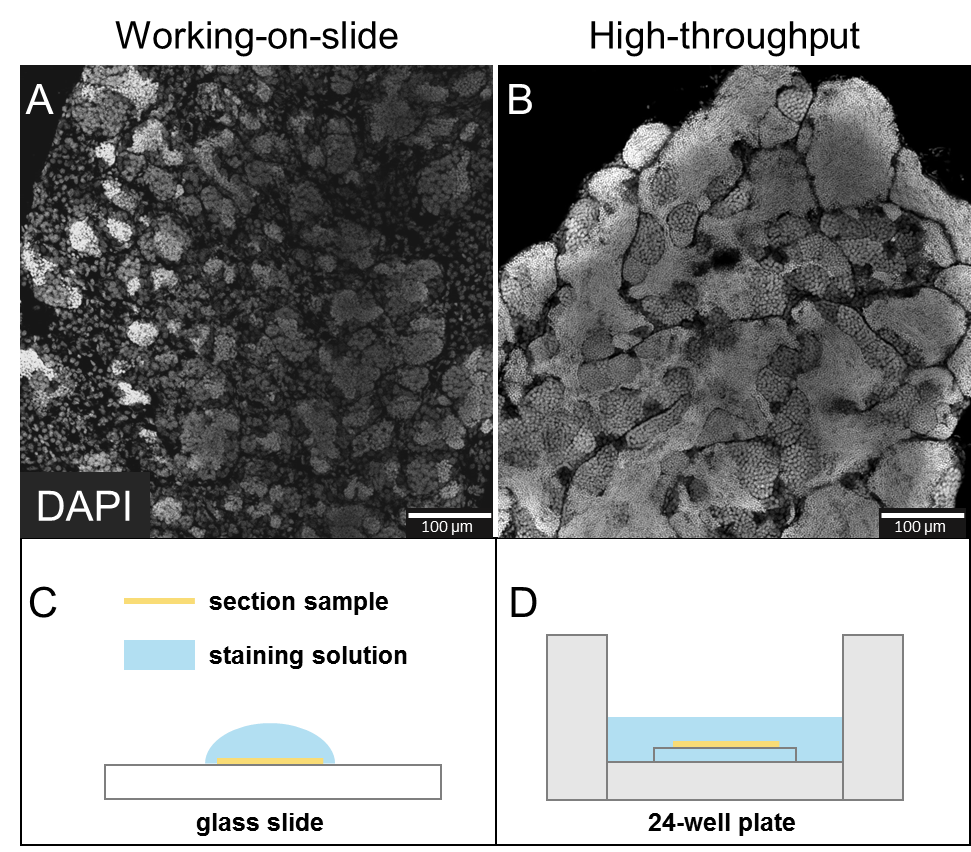
**

**Supplementary Figure 6**

(A) Representative image of uneven staining, which is usually seen in working-on-slide staining. (B) Representative image of even staining, which is usually seen in our high-throughput staining. (C) A schematic diagram showing the side view of staining on glass slide. (D) A schematic diagram showing the side-view of staining in 24-well plate.

**References**

1. Hu X, Li H, Lin Y, Wang Z, Feng H, Zhou M, et al. Genomic Deciphering of Sex Determination and Unique Immune System of a Potential Model Species Rare Minnow (Gobiocypris Rarus). *Sci Adv* (2022) 8(5):eabl7253. Epub 2022/02/03. doi: 10.1126/sciadv.abl7253.
